# Clinical Outcomes of Lumbar Fusions Using Transfacet Screws

**DOI:** 10.7759/cureus.102142

**Published:** 2026-01-23

**Authors:** Iyooh Uche Davidson, Emily C Courtois, Isador Lieberman, Donna D Ohnmeiss

**Affiliations:** 1 Spine Surgery, Texas Back Institute, Plano, USA; 2 Research, Texas Back Institute, Plano, USA; 3 Orthopedics, Texas Back Institute, Plano, USA; 4 Orthopedics, Texas Back Institute Research Foundation, Plano, USA

**Keywords:** lumbar spine surgery, minimally-invasive spine, orthopedic spine surgery, transfacet screw, translaminar screw

## Abstract

Introduction: Posterior lumbar fusion is a widely used technique for stabilizing the lumbar spine, yet complications such as cranial facet violations, increased blood loss, and soft tissue trauma have prompted interest in minimally invasive alternatives. Transfacet screw fixation has emerged as a promising technique, offering advantages such as reduced surgical morbidity, lower risk of adjacent segment degeneration, and greater biomechanical stability in specific patient populations. This study investigates the clinical outcomes of patients undergoing one- or two-level lumbar fusion using transfacet screws.

Methods: A retrospective cohort study was conducted on a consecutive series of 31 patients who underwent posterior lumbar fusion using transfacet screws between 2010 and 2022. Patients were included if they received transfacet or translaminar screw fixation at one or two spinal levels and had a minimum of one-year follow-up. Clinical outcomes were evaluated using Oswestry Disability Index (ODI) scores, visual analog scale (VAS) scores for back and leg pain, and clinical data on reoperation rates and indications were evaluated.

Results: The mean age of the study population was 53.3 years (range 26-83), and 54.8% were male. Most patients (71%) involved a combined interbody and posterior fusion. A total of 82 screws were implanted across 41 levels, with 48 being translaminar facet screws and 34 being transfacet screws. Patients experienced significant improvements in ODI (40.1 vs. 23.1; p<0.001), back pain (6.4 vs. 2.7; p<0.001), and leg pain (4.7 vs. 2.3; p=0.003) scores. The overall reoperation rate was 19.4% (n=6), with half of these reoperations addressing preexisting pathology rather than surgical failure. Five of the six reoperations (83.3%) involved the use of transfacet screws at additional levels. Time from index to reoperation was shorter on average for continued symptomatic patients (19.7 months) than those who developed symptoms due to new pathologies (49.7 months).

Conclusion: This study found that the use of transfacet screw fixation provides a viable alternative to traditional pedicle screws, offering significant pain relief and functional improvement.

## Introduction

Posterior lumbar fusion is a cornerstone of spinal stabilization, frequently employed to manage degenerative spinal conditions, instability, and deformities. Traditional fixation techniques, such as pedicle screw-rod constructs, have demonstrated high fusion success rates but are associated with complications, including increased operative time, excessive blood loss, soft tissue disruption, and increased risk of adjacent segment degeneration (ASD) [1,2]. These concerns have fueled the development of minimally invasive approaches aimed at reducing morbidity while maintaining biomechanical stability.

Various methods of transfacet screw fixation in the lumbar spine were introduced in the 1940s and 1950s as effective spinal fixation techniques [3-5]. These were described as providing enough stability to the spine to reduce or eliminate extended bed rest, casting, or bracing while bony fusion occurred at the operated segment(s). Magerl published on translaminar facet screws in 1984, which is credited as the first description of this technique [6]. During the 1980s, transfacet screws were surpassed by the rapid acceptance of pedicle screws and rods to stabilize the spine, promoting incorporation of the fusion. The transfacet screw technique remains a viable alternative to pedicle screws and rods due to its minimally invasive nature, requiring less soft tissue dissection, and reducing intraoperative complications [7].

The less invasive nature of transfacet screws compared with pedicle screw-rod constructs may have given rise to concerns about the degree of stability they impart, as the construct involves only two screws per treated level versus four pedicle screws and two rods per treated level. There have been several biomechanical studies comparing the stability provided by these transfacet screws to pedicle screws and rods in the lumbar spine. Such studies have generally found that transfacet screws provide similar stability to bilateral pedicle screw-rod constructs [8-10]. However, one study reported that the stability between these constructs was similar only when used as supplemental fixation to a lumbar interbody fusion [11].

Transfacet screws may be advantageous in revision surgeries where anatomical distortions or instrumentation from previous procedures might complicate the use of pedicle screws. Additionally, they may be particularly beneficial for patients with osteoporosis, where pedicle screws have a higher risk of loosening due to poor bone quality [12].

Despite increasing utilization, clinical outcome data on transfacet screw fixation remain limited. While biomechanical and small-scale clinical studies suggest promising results, larger retrospective analyses evaluating patient-reported outcomes, pain relief, and reoperation rates are needed to determine the long-term efficacy of this technique. This study investigates clinical outcomes as measured by functional outcome scores, pain scores, and reoperation rates following one- to two-level lumbar fusion using transfacet screws.

## Materials and methods

Study design

This is a retrospective study that was reviewed by the Institutional Review Board and deemed exempt.

The surgical records from a multi-site spine specialty center were obtained from September 2010 to August 2022 via a surgical case list and further refined with International Classification of Diseases (ICD) codes to identify a consecutive series of patients who underwent lumbar fusion surgery using transfacet screws. Patients received transfacet screws, translaminar facet screws, or a combination of both. The insertion of at least one transfacet screw was designated the index procedure for this study.

Participants

Patients were included if they: 1) underwent a posterior lumbar facet fusion utilizing transfacet screws or translaminar facet screws, 2) only had surgery performed on either 1 or 2 contiguous levels, 3) had at least one year of follow-up, and 4) were at least 18 years of age. Patients were excluded if they: 1) previously underwent a lumbar fusion, total disc replacement, or vertebroplasty, 2) were diagnosed with a trauma, an infection, or a spinal deformity requiring surgical correction, or 3) had an active cancer diagnosis at the time of surgery.

Data collection

For eligible patients, clinic charts were reviewed to collect general descriptive data (age, sex, height, weight). Operative reports were reviewed to determine level(s) operated, estimated intraoperative blood loss, surgical complications, and type of screws implanted (translaminar facet or transfacet screws).

All intraoperative and postoperative complications were recorded. Clinical outcomes such as scores from the Oswestry Disability Index (ODI) as well as back and leg pain using the visual analog scale (VAS) were recorded preoperatively and at the most recent office visit postoperatively. Postoperative data collection included occurrence of screw removal and length of follow-up.

Reoperation was defined as any return to the operating room for addressing the lumbar spine at any timeframe. If a patient required reoperation, the details regarding the reason for reoperation, levels operated, instrumentation removal, or use of additional fixation were recorded. For each case involving screw removal, the reason for removal, time interval from index surgery, and new fixation strategy were recorded.

One independent researcher refined the participant list, and this same researcher performed all data analyses.

Statistical analysis

Descriptive statistics were used to summarize baseline characteristics. Continuous variables were reported as means with standard deviations, and categorical variables were expressed as counts and percentages. Pre- and post-operative changes in ODI and VAS scores were analyzed using the paired Wilcoxon signed-rank test due to non-normal data distribution. This test used complete-case analysis. The effect of age, gender, and BMI on outcomes and reoperations was not explored, as no multivariate analysis was performed on this small cohort. Categorical variables were not compared. Statistical significance was defined as p < 0.05. All analyses were performed using R statistical software (R Foundation for Statistical Computing, Vienna, Austria) [13].

## Results

Patient characteristics

A total of 31 patients met the inclusion criteria (Table 1). Two patients were excluded due to insufficient follow-up (<12 months). The study population consisted of individuals treated for lumbar degenerative conditions, including stenosis, spondylolisthesis, facet hypertrophy, and disc degeneration. The mean patient age was 53.3 years (range: 26 to 83) with an equal distribution of male patients (54.8%) and female patients (45.2%).

**Table 1:** Description of the study population BMI indicates body mass index; SD indicates standard deviation

Characteristics	Value
Total Patients (N)	31
Patient Characteristics	
Male, vs. Female (N, %)	17 (54.8%)
Age, years (Mean, SD)	53.3 (17.6)
BMI, kg/m^2^ (Mean, SD)	27.7 (4.3)

Surgical data and fixation type

A total of 82 screws were implanted across 41 spinal levels (Table 2). Translaminar facet screws were used in 48 instances, while transfacet screws were utilized in 34 cases. Among the procedures, transfacet screws were more frequently employed in combination with interbody fusion techniques (22 cases) rather than as standalone posterior fixation (nine cases). Most of the participants who had an interbody fusion technique in addition to the posterior transfacet screw fixation involved an anterior lumbar interbody fusion (ALIF).

**Table 2:** Index intra-operative data 360 indicates a combination of interbody fusion and posterior approach lumbar surgery

Index Intraoperative Data Summary	Value
Total Screws Implanted (N)	82
Translaminar Facet Screws (N)	48
Transfacet Screws (N)	34
Total Levels Operated (N)	41
Estimated Blood Loss, mL (Mean, SD)	57.5 (47.8)
Complications (N, %)	0 (0.0%)
Use in Combined 360° Fusion, (N, %)	22 (71%)

Transfacet screws were most commonly implanted at L4-L5 and L5-S1 (36 out of 41 levels), with limited use at L3-L4 (four cases) and L2-L3 (one case).

Clinical outcomes

At a mean follow-up of 45 months (range 12-129 months), patients exhibited statistically significant improvements in functional and pain scores (Table 3).

**Table 3:** Comparison of pre- to post-operative scores on outcome assessments *Denotes statistical significance (p<0.05). CI is the confidence interval. ODI: Oswestry Disability Index

	Preoperative (Mean, SD)	Postoperative (Mean, SD)	P-value	Median and 95% CI for Pre- to Post-operative Difference
ODI	40.1 (14.4)	23.1 (15.8)	<0.001*	19.0 [9.65, 29.0]
Back Pain	6.4 (2.4)	2.7 (3.1)	<0.001*	3.9 [2.30, 5.35]
Leg Pain	4.7 (2.7)	2.3 (3.1)	0.003*	3.1 [1.50, 4.50]

Reoperations and adjacent segment disease

A total of six patients (19.4%) underwent a lumbar reoperation (Table 4). The reoperation causes fell into two primary categories: residual pathology (3/6, 50%) and new pathology (3/6, 50%). Three reoperations were performed to address residual pathology of preexisting degenerative conditions requiring further surgical intervention. The remaining three reoperations addressing new pathology were performed primarily for ASD or instrumentation-related complications (painful and loosened transfacet screws at 1 of 2 operated levels). Notably, no cases of pseudarthrosis or screw breakage.

**Table 4:** Reoperation summary 360 fusion is an anterior and posterior lumbar staged operation. Reop is a lumbar reoperation. ALIF is anterior lumbar interbody fusion. LLIF is lateral lumbar interbody fusion. F is female and M is male. *Denotes implanting more transfacet or translaminar screws during the reop.

Patient ID	Age (year) at Index Surgery and Sex	Reason for Index Surgery	Index Surgery Level(s)	Reop Levels	Was a Screw Removed?	Reason for Reop	Reop Surgery/Operative Course	Months from Index to Reop
1	33F	Disc Degeneration and Facet Hypertrophy	L4-S1	L4-L5	Yes	Loosened and Painful Screws	Replacement of L4-L5 Screws using Larger Transfacet Screws*	42
2	60F	Spondylolisthesis	L4-S1	L3-L4	No	Adjacent Segment Disease	LLIF using Transfacet Screws*	66
3	31M	Disc Degeneration	L4-L5	L5-S1	No	Addressing Prior Disc Degeneration	360 (ALIF) using Transfacet Screws*	31
4	44M	Disc Degeneration	L5-S1	L4-L5	No	Adjacent Segment Disease	ALIF (pedicle screws)	41
5	35M	Disc Degeneration	L5-S1	L4-L5	No	Addressing Prior Disc Degeneration	LLIF using Transfacet Screws*	7
6	79M	Spondylolisthesis and Stenosis	L4-L5	L3-L5	Yes	Addressing Prior Stenosis	Screw Removal at L4-L5; Implant Transfacet Screws at L3-L4*	21

Average time from index to reoperation was shorter on average for patients with residual pathology (19.7 months) than for patients with new pathologies (49.7 months).

## Discussion

This study evaluates the clinical and surgical outcomes of lumbar transfacet screws in a cohort of 31 patients with a mean follow-up duration of almost four years. These results indicate that transfacet screws can provide significant clinical improvements in patients undergoing posterior lumbar fusion.

The significant improvements in ODI, back pain, and leg pain scores suggest that transfacet screws are effective in alleviating pain and improving functional capacity. These reductions are comparable to those observed in traditional pedicle screw-based lumbar fusion, where mean ODI improvements range from 18 to 25 points, and postoperative ODI scores average between 20 and 30 [14,15].

One of the most notable proposed advantages of transfacet screw fixation is its minimally invasive nature, which is reflected in the significantly lower estimated blood loss observed in this study. The mean intraoperative blood loss was 57.5 ± 47.8 mL, comparatively lower than in traditional pedicle screw-based fusion, where blood loss is typically reported in ranges between 150 and 500 mL (p < 0.001) [10,16]. This finding corroborates previous studies that highlight the benefits of transfacet screws in reducing surgical morbidity, soft tissue dissection, and operative time [17]. Reduced blood loss and tissue disruption may also contribute to faster postoperative recovery, which is particularly beneficial in older or osteoporotic patients who may not tolerate extensive surgical trauma.

The reoperation rate of 19.4% (6/31 patients) observed in this study falls within the higher end of the range reported for lumbar fusion procedures using pedicle screws, which has been documented to vary between 10% and 25%, depending on patient comorbidities, follow-up duration, and the number of levels fused [1,2]. Importantly, three of the six reoperations (50%) were performed to address preexisting pathology rather than implant-related failure.

Among the six patients who required reoperation, two cases (9.7%) were attributed to ASD. The ASD-related reoperation rate of 9.7% in this study is comparable to the 10.8% to 3.3% rates reported for transfacet screw-based fusions [18,19]. These are known risks associated with lumbar fusion procedures, as mechanical stress may transfer to adjacent levels and poor bone quality may increase risk for screw loosening. Some studies suggest that transfacet screws may have an advantage in this regard, as their smaller implant profile and reduced disruption of posterior spinal elements could result in lower rates of ASD progression [20,21]. Future studies with larger sample sizes and longer follow-up durations could further clarify their impact on ASD.

In addition to ASD, one patient (3.2%) required revision surgery due to symptomatic screw loosening, necessitating the removal and replacement of the existing screws with larger transfacet screws to improve fixation. This screw loosening rate was significantly lower than the 10-15% rates reported for pedicle screws in the literature [10,12]. This lower rate could be attributed to the biomechanical properties of transfacet screws, which engage the cortical bone rather than relying on cancellous bone fixation, as is the case with pedicle screws [22]. Cortical bone engagement provides enhanced screw purchase and may contribute to reduced rates of screw loosening, particularly in patients with poor bone quality.

Notably, five out of the six reoperations (83.3%) involved the implantation of additional transfacet screws. The continued use of transfacet screws in reoperations for ASD, degenerative progression, and screw replacement suggests that they remain a viable fixation method even after prior fusion surgery.

While not a novel spine surgery technique, there has been a resurgence in transfacet-based devices. Studies indicate that transfacet screws offer comparable biomechanical stability to pedicle screws, particularly in short-segment lumbar fusions [9,10]. Evidence also suggests that when compared to pedicle screws, transfacet screws yield comparable complication rates [23,24]. Cortical bone trajectory screws have been associated with higher rates of screw loosening (12.5%) compared to the 3.2% loosening rate observed in this study (p = 0.01) [7]. Similarly, standalone anterior and lateral lumbar interbody fusion techniques, such as ALIF and oblique lumbar interbody fusion, often require additional posterior fixation to enhance stability [25]. Transfacet screws can supplement these techniques, reducing the need for pedicle screw constructs while maintaining adequate posterior fixation (p = 0.03) [15,26,27].

In comparison to the other techniques, incorrect trajectory or transfacet screw misplacement can lead to complications such as facet joint violation or nerve injury, although some authors suggest that the transfacet screw trajectory actually reduces the risk of nerve injury and breach of the spinal canal [20].

Several limitations of this study should be acknowledged. The retrospective design limits control over potential confounding factors, such as variations in screw trajectory and patient comorbidities. Additionally, the sample size of this cohort was restricted to 31 patients, and the majority received the transfacet screws in combination with an interbody fusion anterior to the spinal column, leaving 29% of this cohort only receiving the transfacet screws. This highlights the heterogeneity in this sample; however, this also represents a real-world clinical experience. Further prospective, randomized controlled trials with long-term outcomes comparing transfacet screws to pedicle screw constructs are needed to provide definitive evidence regarding their efficacy, safety, and cost-effectiveness. Longer follow-up, beyond the 45.5-month follow-up recorded in the current study, may also be beneficial for full assessment of ASD after this spinal surgery. Radiographic assessments using computed tomography could provide more objective measures of fusion success and further elucidate the biomechanical properties of transfacet screws.

## Conclusions

This study found that the use of transfacet screws produced significant improvement in pain and self-reported function. Our results suggest a trend towards a favorable safety profile, with low failure rates and minimally invasive nature of implantation. While the findings from this study are encouraging, further prospective, long-term studies are needed to confirm their long-term efficacy, optimize surgical indications, and refine patient selection criteria for clinical implementation in lumbar spinal surgery.
